# Analogies between HPV Behavior in Oral and Vaginal Cavity: Narrative Review on the Current Evidence in the Literature

**DOI:** 10.3390/jcm13051429

**Published:** 2024-03-01

**Authors:** Miriam Dellino, Grazia Pinto, Antonio D’Amato, Francesco Barbara, Francesco Di Gennaro, Annalisa Saracino, Antonio Simone Laganà, Antonella Vimercati, Antonio Malvasi, Vito Maurizio Malvasi, Ettore Cicinelli, Amerigo Vitagliano, Eliano Cascardi, Vincenzo Pinto

**Affiliations:** 1 1st Unit of Obstetrics and Gynecology, Department of Interdisciplinary Medicine, University of Bari, 70124 Bari, Italy; miriamdellino@hotmail.it (M.D.); antonella.vimercati@uniba.it (A.V.); antoniomalvasi@gmail.com (A.M.); ettore.cicinelli@uniba.it (E.C.); amerigo.vitagliano@gmail.com (A.V.); pintov@libero.it (V.P.); 2 Dentistry Unit, Department of Interdisciplinary Medicine, University of Bari Medical School, 70124 Bari, Italy; g.pinto31@studenti.uniba.it; 3 Unit of Otolaryngology, Department of Ophtalmology and Otolaryngology, University of Bari, 70124 Bari, Italy; francescobarbara89@gmail.com; 4 Clinic of Infectious Diseases, Department of Precision and Regenerative Medicine and Ionian Area, Polyclinic of Bari, University Hospital Polyclinic, University of Bari, Piazza Giulio Cesare n. 11, 70124 Bari, Italy; francesco.digennaro1@uniba.it (F.D.G.); annalisa.saracino@uniba.it (A.S.); 5 Unit of Obstetrics and Gynecology, “Paolo Giaccone” Hospital, Department of Health Promotion, Mother and Child Care, Internal Medicine and Medical Specialties (PROMISE), University of Palermo, 90127 Palermo, Italy; antoniosimone.lagana@unipa.it; 6 Dentistry Faculty, Sapienza University of Rome, 00161 Rome, Italy; vitomalvasi7@gmail.com; 7 Pathology Unit, Department of Precision and Regenerative Medicine and Ionian Area (DiMePRe-J), University of Bari, Piazza Giulio Cesare 11, 70121 Bari, Italy; eliano20@hotmail.it

**Keywords:** human papilloma virus, oral cavity infection, genital infection, HPV-related cancer, squamous cell carcinoma, oral cancer, cervical cancer

## Abstract

Human genital papilloma virus infection is the most prevalent sexually transmitted infection in the world. It is estimated that more than 75% of sexually active women contract this infection in their lifetime. In 80% of young women, there is the clearance of the virus within 18–24 months. In developed countries, oral squamous cell carcinoma (OSCC) is now the most frequent human papilloma virus (HPV)-related cancer, having surpassed cervical cancer, and it is predicted that by 2030 most squamous cell carcinomas will be the HPV-related rather than non-HPV-related form. However, there are currently no screening programs for oral cavity infection. While the natural history of HPV infection in the cervix is well known, in the oropharynx, it is not entirely clear. Furthermore, the prevalence of HPV in the oropharynx is unknown. Published studies have found wide-ranging prevalence estimates of 2.6% to 50%. There are also conflicting results regarding the percentage of women presenting the same type of HPV at two mucosal sites, ranging from 0 to 60%. Additionally, the question arises as to whether oral infection can develop from genital HPV infection, through oral and genital contact or by self-inoculation, or whether it should be considered an independent event. However, there is still no consensus on these topics, nor on the relationship between genital and oral HPV infections. Therefore, this literature review aims to evaluate whether there is evidence of a connection between oral and cervical HPV, while also endorsing the usefulness of the screening of oral infection in patients with high-risk cervical HPV as a means of facilitating the diagnosis and early management of HPV-related oral lesions. Finally, this review emphasizes the recommendation for the use of the HPV vaccines in primary prevention in the male and female population as the most effective means of successfully counteracting the increasing incidence of OSCC to date.

## 1. Introduction

Human papilloma virus (HPV) infections have a high incidence in the general population, with genital HPV infection being the most prevalent sexually transmitted infection in the world [1]. It is estimated that more than 75% of sexually active women contract it in their lifetime [2]. In Europe and North America, its peak prevalence is recorded in young women under the age of 25, soon after the initiation of sexual activity. In 80% of young women, however, the virus clears within 18–24 months [3]. The persistence of high-risk genotypes can identify women at risk of preneoplastic lesions, which can potentially progress to cervical cancer [4]. Particularly on the cervix, the presence of the metaplasia zone represents an active cellular tissue, ideal for the HPV replication cycle. Indeed, HPV requires the differentiation and replication of the host cell for its own viral replication and survival [5,6,7,8,9]. Beyond the cervix, HPV also reveals its oncogenic potential in other organs, including the oropharynx, anus, vulva, vagina, and penis [10]. Cervical cancer frequently develops through a sequence of premalignant lesions, described as different grades of dysplasia. Although HPV is widespread, the literature shows that only a small number of women have a recurrent HPV infection and subsequently develop precancerous lesions. Indeed, several studies have described spontaneous HPV clearance, estimated at approximately 20–30% of cases at three months, almost 50% at six months, and nearly 60–70% at one year. Consequently, several studies report that over 90% of HPV infections and infection-induced lesions are transitory and resolve spontaneously [11]. 

The ability of each organism to eliminate the virus may depend on several factors, among which, concomitant vaginal infections, a local microflora imbalance, and immune response defects seem to be decisive [12]. For this reason, screening makes it possible to identify patients at risk and to implement a prevention programme [3].

Indeed, the implementation of national screening programs, complemented by the introduction of the anti-HPV vaccine, has succeeded in lowering HPV morbidity and cervical cancer mortality by 70% (7.4 per 100,000) in industrialized countries [13]. In contrast to cervical cancer, the natural history of HPV infection in the oropharynx remains inadequately understood [14].

Indeed, studies highlight considerable variability in the rate of viral clearance, reporting a median time to elimination ranging from 6 to 18 months [15]. A question arises regarding the connection between oral and genital HPV infection: it remains unclear as to whether oral infection can arise through oral and genital contact, by self-inoculation, or whether it should be considered an independent event [15].

Additionally, it has been observed that certain risk factors, such as smoking, number of partners, age, and sexual habits can impact the potential presence of the virus in the oropharynx of women [16]. The diagnosis and treatment of oral squamous cell carcinoma (OSCC) have been associated with a significant decrease in the frequency of vaginal and oral sex, regardless of tumor HPV status; a decline in sexual behavior is observed in both HPV-positive and HPV-negative oropharyngeal cancer patients [16]. The very similar decline in the frequency of sexual behaviors in HPV-positive and HPV-negative patients would suggest that knowledge and concern about the sexual transmission of HPV is not the sole reason behind changes in sexual behavior [17]. While sexual abstinence is undeniably the most reliable method of protection against HPV, it cannot be considered a panacea for preventing HPV and other sexually transmitted infections [17]. Therefore, adopting a vigilant approach to sexual practices becomes a crucial recommendation [18]. We also need to deal with the new, emerging evidence that in developed countries, OSCC has become the most frequent HPV-related cancer, surpassing cervical cancer [19]. Furthermore, it is predicted that by 2030, most squamous cell carcinomas will be HPV-related rather than the non-HPV-related form [16]. An important aspect to consider in HPV transmission is oral concordance, defined as the simultaneous appearance of the same type of HPV at different anatomical sites [20]. HPV transmission and genotype concordance between heterosexual partners showed HPV-DNA concordance in 87.5% of couples [20]. However, the prevalence of oral HPV infections with concomitant cervical HPV infection shows contradictory results, with some authors reporting high-prevalence and others not. Long-term positivity (over 24 months) of high-risk cervical and oral HPV may induce an increase in cervical and oral cancer with stratified risk according to genetic background and HPV subtype [21]. Notably, oral HPV16 DNA is commonly detected among patients with HPV-OSCC at diagnosis, yet the prevalence rate among their partners is confusing [22]. Heterosexual partners of patients with OSCC exhibit an estimated oral HPV prevalence rate of approximately 15%, with a concordance for their HPV genotype of 49% [23]. The evidence that male sexual partners of women with cervical cancer have approximately twice the risk of tonsillar cancer supports the theory of oral transmission of HPV through orogenital sex [23]. 

Despite these findings, and the fact that these types of cancer have a prognosis closely related to diagnostic timing, currently, oral tumor screening is not carried out [24]. This is because early diagnosis is considered nearly impossible due to the frequent presence of HPV, even in the absence of visible lesions in the oral cavity [1]. In light of this, the main objective of our study was to evaluate the potential connection between the presence of HPV in both the oral and genital mucous membranes in women with HPV lesions at the lower genital tract. This assessment was conducted through an analysis of the existing literature data, aiming to identify the presence of a population at risk for lesions of the oral cavity within the subset of women with HPV at high risk for cervical lesions. The end goal is to be able to propose a targeted screening of HPV-related oral lesions in the at-risk population, facilitating the early diagnosis of oral lesions. 

## 2. Oral–Cervical–Perinatal HPV Transmission

**Figure 1:**
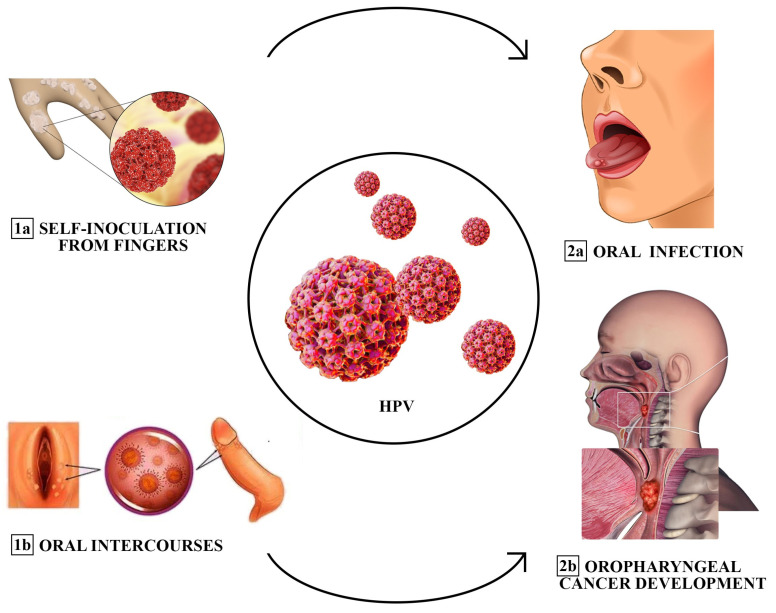
Different transmission modes of oral HPV infection (sequentially ordered: from 1a and 1b to 2a and 2b). Fingers have been described as a source of transmission or self-inoculation of HPV to the oral cavity. Oral sex could be another predominant cause of infection among adults. Risk factors associated with the development of the infection include promiscuity, prior diagnosis of sexually transmitted diseases (STDs), and a high frequency of oral intercourse.

The extragenital sites, the oral and the anal area, each exhibit distinct probabilities of transmission, influenced by factors such as sexual relationships, duration of sexual relations, and the specific number of different acts within a partnership [25]. Additionally, fingers have been identified as a possible source of transmission or self-inoculation of HPV to the oral cavity [25]. In men, oral HPV infection is significantly correlated with urinary HPV infection, with HPV16 being the most common type [20]. Similarly, the majority of female patients have concordant oral and urinary HPV types [20]. With regard to oral–genital transmission, although HPV is known to have the potential to affect the intimate lives of women and their partners, it has rarely been explored in the literature [16]. In fact, oral transmission of HPV and, consequently, the risk of oral cancer, increases in women with cervical cancer and their spouses [16]; this finding suggests a cross-transmission between the mouth and genitals. However, data in this regard are discordant, with only a few studies reporting concordant rates of oral and cervical HPV infection above 65% [26] (Figure 1). Among these, a recent study in Italian women compared the prevalence and concordance of HPV infections between the oropharynx and genital sites, reporting that the prevalence of oral HPV infections was high among women with concomitant genital HPV infections (22%) compared to genital HPV-negative women (0%) [26]. HPV16 was the most common high-risk genotype at both sites, and there was low concordance between HPV genotypes at the two anatomical sites (kappa = 0.125) [16]. On the other hand, other studies report that the overall prevalence of oral HPV infections is varied, with values ranging from 0.2% to 20.7%, and rates of concomitant infections with oral and cervical HPV are reported in less than 10% of cases [16]. The variability of these data can be attributed to different inclusion criteria, clinical contexts, sampling methods, and detection tests. In none of the women with oral HPV did we find relevant clinical lesions [16]. Therefore, an oral examination alone cannot exclude the possibility of an oral HPV infection, which is primarily subclinical. In addition, a multivariate analysis showed that oral infection in women with HPV-positive genitalia was significantly related to age and sexual habits, particularly oral sex [27]. In fact, it is highlighted that there is a higher incidence of HPV in the oral cavity in women aged between 36 and 50 years compared to patients under the age of 25 years [28]. Regarding the frequency of sexual intercourse, the probability of being positive for oral HPV increased by 77% if women had sexual intercourse more than 10 times a month (95% CI: 2.67, 2223.04), compared to those who had intercourse 0–1 times [16]. A study by Hemminki K et al. suggested a significant association between oral sex and oral HPV; among women who engaged in sexual activity “occasionally”, there was an 18.29% increase in the chances of testing positive for oral HPV compared to those who did not [29]. Furthermore, husbands of women with cervical cancer have two times the risk of tonsillar cancer. Transmission dynamics are influenced by sex, with female genitalia having a higher rate of transmission than male genitalia [30]. The keratinized penis epithelium is more resistant to HPV infection, and the amount of biological fluid that reaches the oropharynx can also be a factor in this gender difference [30]. Finally, it appears that women develop a stronger systemic immune response than men after a genital HPV infection [31]. This would more efficiently protect women than men in the case of subsequent exposure to HPV. In women aged 18 to 69 years, data from oral and cervicovaginal HPV DNA screening showed that cervicovaginal HPV infection was present in 45.2%, oral HPV infection in 4.1%, double infection in 3.0%, and concordant infection in 1.1%. Nearly half of U.S. men have HPV infection of the penis [31]. High burden of HPV infection of the penis has been associated with oral HPV infection. In cases of sexual intercourse with only one homosexual female partner, the risk of oral HR-HPV infection was 19.3% if the partner had a genital infection, increasing to 22.2% if oral sex was performed with two or more homosexual female partners. The incidence of oral HPV among young heterosexual men was lower than in same-sex relationships yet increased with a greater number of partners (17.9%), in those who engaged in oral sex (28.6%), and in partners with oral or genital infections (11.5%) [32]. In addition, prevalence increased with the frequency of oral sex among men whose partner had a genital infection with the same type of HPV, supporting evidence that oral HPV can be transmitted orally–orally or orally–genitally [32]. In fact, studies report how the low overall prevalence of specific genotype concordance across anatomical sites can be explained by variation in susceptibility to HPV infection, differences in exposure characteristics, and disparities in the natural history at each mucosal site [33]. Additionally, other carriers of infection and the frequency and early age of oral sex appears to contribute significantly to the probabilities of HPV-OSCC [33]. Notably, men and women with genital wart-like lesions represent a high-risk population, with 64.6% of patients with condyloma acuminata showing HPV types associated with an increased risk of dysplasia. The vast majority of these patients had HPV6 DNA detected at the wart site and also at other sites [20].

## 3. Cervical HPV and Perinatal Transmission

Low-risk (LR)-HPV-related diseases, such as the recurrent development of respiratory papillomatosis (RRP), can occur particularly in infants of patients with condyloma [34]. It can be transmitted in utero, via transplacental or ascending infection, or during the passage through an infected birth canal. Caesarean section does not fully protect newborns from HPV [35]. HPV, including high-risk types 16 and 18, has also been detected in tonsillar or adenoid samples from children with tonsillar or adenoid hyperplasia, chronic tonsillitis, and normal mucosa [36]. Given the finding of the high prevalence (44.9%) of HPV in prenatal tests in young pregnant women, HPV tests were performed on the placenta and the newborn with a presence of 14% in placenta samples and 11.2% in the oral cavity of the newborns [36]. Although the exact mode of transmission is not well understood, the oral prevalence of HPV ranged from 10% to 30% among infants born to HPV-positive mothers [37]. Vertical transmission can occur in utero through ascending infection (transplacental transmission), during vaginal delivery, and even via caesarean section, while transmission immediately after delivery can occur through hand-to-hand contact. HPV can also be transmitted to newborns through breastfeeding. Notably, the high rates of HPV DNA transport detected in oral samples of newborns gradually decrease during the first 3 years of life. Research has confirmed the association between perinatal transmission of LR-HPV and RRP in neonates at birth [38]. A prospective cohort study evaluating the dynamics of HPV infections in parents and their children reported HPV genotype distribution and virus persistence in maternal oral mucosa in 17% [38]. Children may represent a reservoir of high-risk “silent” HPV types that may be key to HPV persistence and related carcinogenesis in adulthood [39].

## 4. HPV Clearance or Progression

**Figure 2:**
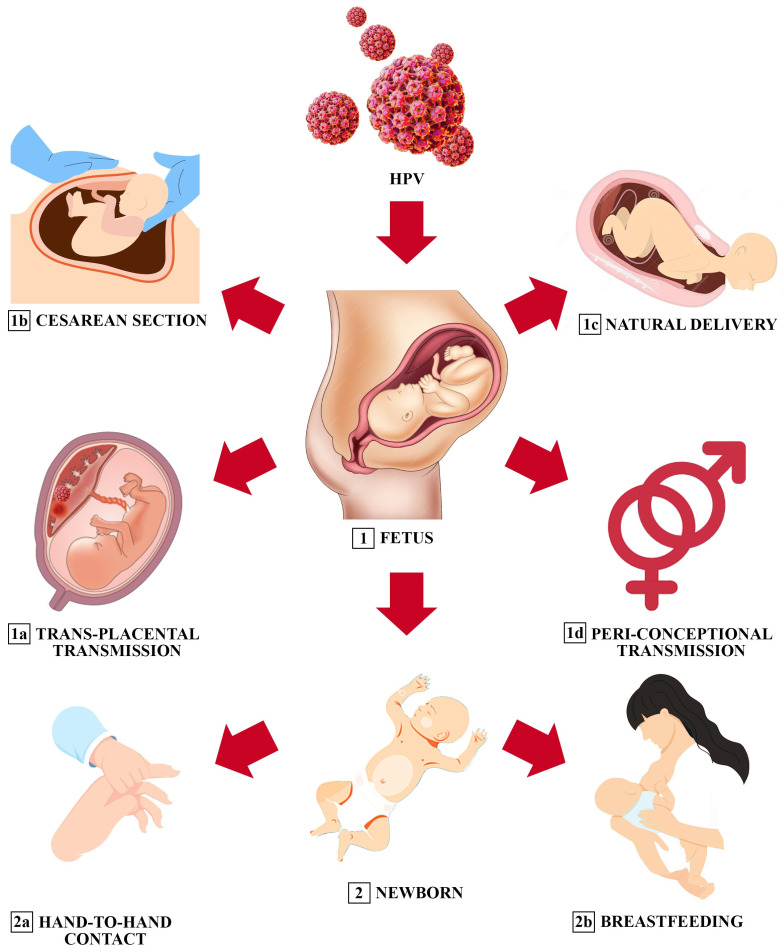
Potential mechanisms of vertical transmission of oral HPV infection (sequentially ordered: from left to right, from 1a to 1d and from 2a to 2b). Infection can occur with transplacental transmission or at the time of delivery. In infants, vertical transmission can occur through breastfeeding or direct contact as they go along. The virus can manifest itself through multiple low-risk HPV (LR-HPV)-related lesions, such as in respiratory papillomatosis, or it can persist in the airways of infants, establishing a significant reservoir of HR-HPV that may increase the risk of carcinogenesis in adulthood.

Our understanding of the natural history of HPV infection has primarily focused on the cervix. In this context, HPV acquisition occurs shortly after sexual debut, with the majority of infections resolving within 1–2 years. While most infections are transient, persistent HR-HPV infection is the leading cause of cervical cancer development [11]. Acquisition of oral oncogenic HPV infection is less common than that of genital infection, although infections at both sites appear to disappear at about the same rate and, in any case, also with both a vertical and non-vertical mechanism (Figure 2). 

**Figure 3:**
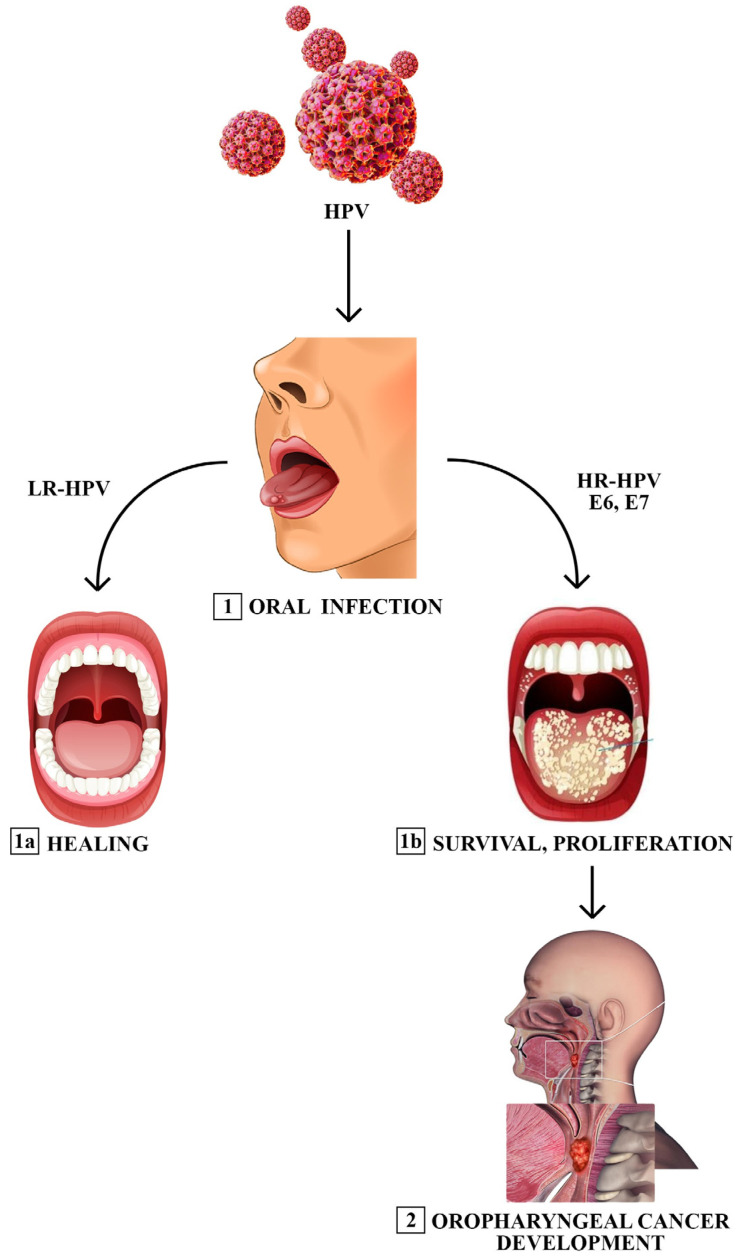
Interaction between virus and host within the oral cavity. Clearance of oral HPV infection may be similar to that of anogenital HPV infections in healthy populations. The spontaneous resolution aligns with the titers of antibodies against HPV and is more frequent in case of an LR-HPV contamination. Conversely, the persistence of the infection into the oral cavity-which is often subclinical–is more frequently associated with high-risk HPV (HR-HPV) genotypes and is the major risk factor for disease progression and oral dysplasia.

Several groups report that the clearance of oral HPV infection is similar to that of anogenital HPV infections in healthy populations. Increased oral viral load was associated with reduced clearance of infection, consistent with the literature on cervical cancer [20]. In a female cohort with HPV16-positive cervical and oral samples, natural clearance correlates with HPV antibody titers. The duration of HPV persistence exceeding 12 months led to an increased risk of disease progression in cervical and oral dysplasia [40]. The time evaluated for neoplastic transformation was reported after a persistence of HPV infection longer than 6 months. The longer the duration of an HPV infection, the greater the likelihood of continued persistence and the greater the likelihood of detecting HPV DNA at any given time [34]. A study in which the patients were followed for >7 years revealed that 18% of HPV16 infections persisted beyond 24 months, potentially conferring a higher risk of HPV-related OSCC (Figure 3).

Clearance rates also vary by virus type, with LR-HPV genotypes being eliminated from the oral mucosa more rapidly than HR-HPV genotypes [41]. The most persistent is HPV16; it has been identified as the most persistent type in the oral cavity, with longer persistence observed among men (22 months) than among women (19 months) [42]. Persistent oral HPV infection has been associated with a low concentration of salivary matrix metalloproteinase-8 (MMP-8), which could indicate a failure in oral anti-inflammatory defense mechanisms [42]. Currently, different authors have questioned the role of protective immunity or immunological fragility to HPV, and the question is still highly debated today, especially regarding the role of both humoral and cell-mediated local immunity as well as protection against HPV infections. This topic is extremely difficult since the majority of HPV infections tend to regress and not evolve into neoplastic lesions. Indeed, it is known that in some women with persistent high-risk HPV infection, a minority of infected cells can ultimately lead to tumor development and neoplastic progression [43,44]. The key to this process is represented by the evasion of the host’s immune surveillance with impaired recognition of the tumor antigen [43,44], as well as the acquired inability to eradicate the viral host usually controlled by the viral locus human leukocyte antigen (HLA) [45,46], comprising molecules with different functions grouped into classes: I (A, B, C, E, F, G), II (DR, DQ, DP), and III [47]. Increasingly clear evidence from the literature has highlighted how HLA molecules orchestrate the immune response in various types of cancer [48,49] by acting on immune cells, altering the functionality of natural killer cells [50,51], of T and B lymphocytes [52], or of macrophages [53]. In different populations, HLA-G polymorphisms are found to be risk factors for HPV infections [54] and correlate with a greater risk of developing high-grade cervical lesions or the tendency to develop pre- and invasive squamous cell forms [55,56,57]. In agreement with that described by Rodriguez et al. [58] via immunohistochemical analysis, Yoon BS et al. also reported that high expression of HLA-G mRNA was correlated with early cervical cancer [59], and at the same time, other authors have highlighted that HLA-G levels are inversely proportional to the percentage of lymphocytes infiltrating the tumor [60]. This finding is strengthened by other manuscripts that have correlated HLA-G expression and poor prognosis [61,62]. These data are consistent with other HLA class I polypeptides [45,46] and with other class II polypeptides [63,64,65,66,67,68,69], but also with patients with squamous cell carcinoma of the head and neck [21,70].

## 5. The Role of Oral and Vaginal Microbiota in Viral Oncogenesis

There is abundant evidence demonstrating how the alteration of vaginal or oral microbiota in patients with high-risk HPV can favor the onset of both cervical and oral cancer, respectively [71]. Particularly, there is evidence that HPV16 can integrate with host-cell DNA and activate oncogenes, and oral dysbiosis and synergistic effects in oral microbial communities may promote cancer development [72]. This is because different microorganisms in the oral cavity interact with each other instead of existing on their own and adhere together to form a microbial community through aggregation and coaggregation. A recent review article lists cancer as one of the comorbidities of periodontal disease; according to a case-control study, patients with periodontitis had 3.7 times the risk of developing oral cancer compared to controls, indicating a potential link between periodontitis and oral tumors [73]. In particular, microbial dysbiosis can act as a bridge between periodontitis and oral tumors. Several studies have shown substantial differences in bacterial diversity and the relative abundance of certain bacteria in the oral flora of cancer patients compared to controls with high-throughput sequencing techniques [74]. Several studies have reported the possibility of using oral swabs to analyze the microbiota on the surface of OSCC lesions and have found an increase in microbial diversity (alpha diversity) [75]. In addition, there was an increase in *Fusobacterium* and a decrease in *Streptococcus* at the tumor site since some authors examined tissue biopsy samples from OSCC patients and found that *Prevotella*, *Corynebacterium*, *Pseudomonas*, *Deinococcus*, and *Noviherbaspirillum* were enriched in tumor tissues, while *Actinomyces*, *Sutterella*, *Stenotrophomonas*, *Anoxybacillus*, and *Serratia* were significantly less abundant. Another study focused on microbiota changes in the saliva of OSCC patients and found that *Prevotella melaninogenica*, *Fusobacterium* sp., *Prevotella pallens*, *Dialister*, *Streptococcus anginosus*, *Prevotella nigrescens*, *Campylobacter ureolyticus*, *Prevotella nanceiensis*, and *Peptostreptococcus anaerobius* showed greater abundance than normal. Another study reports that at the genus level, the abundance of *Fusobacterium periodonticum*, *Parvimonas micra*, *Streptococcus constellatus*, *Haemophilus influenzae*, and *Filifactor alocis* was positively correlated with OSCC progression, while the abundance of *Streptococcus mitis*, *Haemophilus parainfluenzae*, and *Porphyromonas pasteri* was negatively correlated with the progression of OSCC [76]. Consequently, the results obtained from various tissue sources and sampling techniques varied. In terms of bacterial diversity, various sampling strategies produced contradictory results. In saliva and tissue biopsies of OSCC patients, bacterial diversity decreased, but bacterial diversity increased in oropharyngeal swab samples from tumor surfaces [77]. In addition, the oral microbiome alters as the cancer progresses. In fact, some studies found that the abundance of fusobacteria increased steadily with the advancement of cancer from stage 1 (4.35%) to stage 4 (7.92%), compared to 2.98% in healthy controls. Furthermore, age affects the microbiome of OSCC patients.

Yu et al. analyzed the microbiota of 20 older patients (>60 years) and 20 younger patients (50 years) using 16S rRNA sequencing. They found that the younger group was significantly enriched in *Ralstonia*, *Prevotilla*, and *Ochrobactrum,* while *Pedobacter* was more abundant in the older group. This suggests that the role of bacteria in promoting OSCC may differ between older and younger populations [78]. Iron-ion transport, tryptophanase activity, peptidase activity, and superoxide dismutase increase considerably in the microbiota of OSCC patients compared to healthy controls and predict, to some extent, the carcinogenic mechanism [79]. Similarly, Stashenko et al. analyzed the metabolism of oral microbes in mice with OSCC and found that the microbiota was overexpressed in OSCC-associated metabolic pathways, including nitrogen transport, stress response, interspecies interactions, modulation of the Wnt pathway, and amino acid and lipid biosynthesis. Despite the clear link between oral microbiota dysbiosis and oral cancer, a precise mechanism of carcinogenesis has not yet been revealed. The 16s rRNA sequencing has been widely used for the analysis of oral microorganisms in cancer patients and controls [80]. However, due to differences in sampling sites, sampling methods and data processing, sequencing results have been inconsistent between studies, which, to some extent, reduced the reliability of sequencing results [80]. Therefore, a standardized technique is needed in the future to regulate sampling procedures. Chronic infection is considered a risk factor in the development of cancer [81]. Evidence suggests that periodontal pathogens, such as *Porphyromonas gingivalis*, *Fusobacterium nucleatum*, and *Treponema denticola*, are associated with OSCC [81]. They can stimulate tumorigenesis by promoting epithelial cell proliferation while inhibiting apoptosis and regulating the inflammatory microenvironment. Moreover, *P. gingivalis* is a known keystone pathogen related to chronic periodontitis. Clinical studies found that *P. gingivalis* was widely present in gingival squamous cell carcinoma tissue compared to normal gum tissue, suggesting a potential association between *P. gingivalis* and squamous cell gum cancer [82]. Indeed, in a specific oral microbiome, the interactions of microbial communities can also influence oral cancer. Polymicrobial synergy and dysbiosis are the etiology of periodontal disease. Moreover, the development of periodontal biofilm is similar to cancerous human cell communities. Furthermore, research into the mechanism of *T. denticola*-induced cancer is still at a very early stage. Recent research indicates that *T. denticola* directly promotes OSCC cell proliferation, as it has been found to promote OSCC migration by activating crosstalk between TLR/MyD88 and Integrin alfa V/FAK [83]. In short, *T. denticola* and Td-CTLP provide a microenvironment of tumor tissue conducive to invasion and metastasis. *Candida*, especially *Candida albicans*, is a typical oral component. *Candida albicans* is an opportunistic pathogen carried by about 80% of the general population and is harmful to immunocompromised individuals (HIV, cancer, or transplant patients) [84]. Regarding the association between *Candida* and tumors, it is a well-known fact that leukoplakia of the oral mucosa infected with *Candida* is more likely to progress to cancer than uninfected leukoplakia. Although *Candida*, particularly *Candida albicans*, has been highly related to oral cancer based on epidemiological studies, the molecular mechanism underlying *Candida albicans*-induced carcinogenesis remains controversial. Colonization of the host epithelium by *Candida albicans* depends on the imbalance between the virulence components of *Candida albicans* and the host defenses. Some mechanisms have been hypothesized for invasion and colonization of epithelial cells by *Candida albicans* [85]. The first mechanism is catabolic enzymes released by *Candida albicans*, such as aspartic proteases, which can degrade the subendothelial extracellular matrix as well as laminin 332 and E-cadherin, thus allowing the mycelium to penetrate the host cell or migrate through cells [86]. Another mechanism is that *Candida albicans* induces cellular endocytosis by secreting Als3, an invasion that binds to E-cadherin on host cells [87]. Moreover, a recent study determined that *Candida albicans* infection promoted oral cancer incidence in a 4-nitroquinoline 1-oxide (4NQO)-induced mouse tongue carcinogenesis model and promoted OC progression in a tongue tumor-bearing mouse model (C3H/HeN-SCC VII). Indeed, the tumor-associated macrophage (TAMs) infiltration was elevated during *Candida albicans* infection. Meanwhile, the attracted TAMs polarized into M2-like macrophages with high expression of programmed death ligand 1 (PD-L1) and galectin-9 (GAL-9) [87]. In addition, about 10% of *Candida albicans* isolates from the cervix and oral specimens produce gliotoxin and other mycotoxins that target and eliminate T lymphocytes, especially IFN-γ producers [88]. 

## 6. Vaccination Prophylaxis and Cancer Prevention

The implementation of primary prevention aims to reduce the burden of HPV infection and HPV-related disease [89]. There are five approved HPV vaccines, three of them in Europe, which prophylactically prevent HPV infection, despite each of them targeting different strains [90]. With more than 200 million doses administered to date, HPV vaccines are considered to be safe and effective in preventing HPV-related infections and cancers [89]. Therefore, in 2020, the FDA in the United States and the EMA in Europe extended the use of HPV vaccines to males [91]. Moreover, anti-HPV vaccines are also recommended in order to successfully counteract increasing incidence of OSCC [92], although time is needed in order to study the specific benefits of HPV vaccines for OSCC given the long interval between HPV infection and the development of oropharyngeal tumors. On the other hand, HPV vaccines do not increase clearance in patients with ongoing HPV infection [93]. Regarding secondary prevention, surprising data are arising. Indeed, current studies have focused on the role of vaccination after conservative surgery for high-grade cervical dysplasia. In particular, the study by Gherlardi et al. showed that quadrivalent HPV vaccination injected 30 days after conization reduced the risk of subsequent recurrence by 81.2% (95% CI, 34.3–95.7), irrespective of causal HPV type [94]. This does not imply a therapeutic effect of the vaccines but underlines their role as an adjuvant to surgical treatment. Nevertheless, a randomized placebo-controlled study, with a larger number of patients, would be required to confirm these findings. In the meantime, it is important to attract the younger population to improve HPV awareness and to educate them as to the importance of primary prevention to enhance the widespread adoption of HPV vaccination.

## 7. Conclusions

It is now commonly accepted that a persistent HPV infection is a necessary precursor for cervical cancers, as well as for OSCC. Considering that the adoption of barriers in orogenital intercourse is much less widespread than in vaginal intercourse, we believe that it is necessary to recommend preventive measures, suggest changes in sexual behavior, and inform sexual partners of the risk of transmission of HPV to the oral cavity. Therefore, patients with high-risk HPV cervical lesions should be monitored through oral screening protocols, even if the probability of identifying a clinically visible lesion is low. It would be highly appropriate to extend the HPV DNA test to the oral cavity in these women so as also to evaluate the genotypes present in both locations. In this sense, we hope that there will be an ever-increasing number of multi-institutional experimental studies in order to strengthen and expand the data relating to HPV vaccines.

## Data Availability

Data are not available due to the nature of the article (review).
